# Diabetes and pulmonary tuberculosis: a global overview with special focus on the situation in Asian countries with high TB-DM burden

**DOI:** 10.1080/16549716.2016.1264702

**Published:** 2017-01-13

**Authors:** Chunlan Zheng, Minhui Hu, Feng Gao

**Affiliations:** ^a^ Department of Internal Medicine – Section 5, Wuhan Pulmonary Hospital (Wuhan Tuberculosis Control Institute), Wuhan, P.R. China; ^b^ Department of Endocrinology, Union Hospital, Tongji Medical College, Huazhong University of Science and Technology, Wuhan, P.R. China

**Keywords:** Asia, bi-directional screening, diabetes–tuberculosis prevalence, treatment challenges, tuberculosis susceptibility

## Abstract

**Background**: The double burden of tuberculosis (TB) and diabetes mellitus (DM) is hitting certain Asian countries harder than other areas. In a global estimate, 15% of all TB cases could be attributable to DM, with 40% of those cases coming from India and China. Many other countries of South, East, and South-East Asia are of particular concern given their TB burdens, large projected increases in DM prevalence, and population size.

**Objective**: In this narrative review, we aimed to: (i) give an overall insight into the evidence on TB-DM epidemiology from high double burden Asian countries, (ii) present the evidence on bi-directional screening implementation in this region, (iii) discuss possible factors related to higher TB susceptibility of Asian diabetic patients, and (iv) identify TB-DM comorbidity treatment challenges.

**Methods**: The PubMed and Google Scholar databases were searched for all studies addressing DM/TB epidemiology, bi-directional screening and management in South, East and South-East Asia.

**Results**: We identified the DM prevalences among TB patients as ranging from approximately 5% to more than 50%, whereas TB prevalences among diabetic patients were 1.8–9.5 times higher than in the general population in developing Asian countries. Evidence from studies designed to address diagnosis and treatment of the dual disease in these critical regions is scarce as well as the evidence related to possible DM patients’ genetic and acquired predisposition for TB.

**Conclusion**: More prospective studies specifically designed to address adequate screening techniques, identify patients at risk, and define an adequate treatment of dual disease in this region are needed without delay.

## Background

Diabetes mellitus (DM) is a serious lifelong disease that has been increasing in prevalence year after year. In 2012 alone, it caused 1.5 million deaths worldwide. The global prevalence has risen from 4.7% to 8.5% since the 1980s, and it was estimated that 422 million of adults were living with DM in 2014 [1]. This increase has been due to the accelerated rise of DM in low- and middle-income countries rather than in high-income countries [1]. By 2030, it is estimated that approximately 552 million people will be affected by the disease [1]. A national survey study from 2007 to 2008 reported that DM had reached epidemic proportions in China with a prevalence of 9.7% [2].

In contrast to DM, tuberculosis (TB) incidence has been decreasing by an average of 1.5% per year since 2000, to a current level 18% lower than the incidence reported in 2000. However, TB remains one of the world’s biggest threats. In 2014 alone, 1.5 million people died from TB and another 9.6 million are speculated to have fallen sick [3]. Of these, Indonesia and China had the largest number of cases [3]. In addition, there is an even more unsettling problem – multidrug-resistant TB (MDR-TB). These statistics, along with worsening of TB through comorbidities such as HIV and DM, jeopardize the global objective of ending TB by 2030 and achieving its elimination by 2050 [3,4].

Worldwide, there are an estimated 9.6 million new patients with active TB annually [3] and of them, 1 million have both TB and DM (TB-DM) [5]. In the current scenario, the number of patients with TB-DM comorbidity is higher than the number of patients with TB-HIV co-infection around the world [6]. This is extremely worrying, as highlighted in a review by Harries et al.:

The response to the growing HIV-associated TB epidemic in the 1980s and 1990s was slow and uncoordinated, despite clearly articulated warnings about the scale of the forthcoming problem. We must not make the same mistake with diabetes and TB [7].

China has one of the highest TB burdens in the world, including its multidrug resistant forms, along with a growing diabetic population. In China, there are around 1.5 million new TB cases and 270,000 TB deaths each year [8]. China accounts for almost 17% of the world’s burden of TB, and it also has a very large burden of DM with nearly 100 million people affected [8]. In India, in 2011, there were 61.3 million people living with DM. This country also has 1.98 million people developing TB and almost 300,000 people dying of it each year [9]. In Indonesia, every year, 450,000 new TB cases occur resulting in 64,000 TB deaths. Moreover, Indonesia is ranked number 6 in terms of DM cases in the world [10].

Even though awareness about the possible consequences of the dual disease now exists in almost all Asian countries with a high TB-DM burden, there are still many questions to be addressed. This review gives a global overview of epidemiological researches related to TB-DM comorbidity, placing special attention on the TB-DM epidemic in developing Asian countries. It also addresses the question of the susceptibility of Asian diabetic patients to develop TB, provides an insight into the current TB-DM care challenges, and reviews the status of bi-directional screening implementation in Asian countries.

## Methods

The PubMed and Google Scholar databases were searched using the following English-language terms: ‘tuberculosis’ and ‘diabetes’ and/or ‘south east Asia’ and/or ‘south Asia’ and/or ‘east Asia’ or the following countries, ‘Afghanistan,’ ‘Bangladesh,’ ‘Cambodia,’ ‘China,’ ‘India,’ ‘Indonesia,’ ‘Korea,’ ‘Laos,’ ‘Malaysia,’ ‘Nepal,’ ‘Pakistan,’ ‘Singapore,’ ‘Sri Lanka,’ ‘Thailand,’ or ‘Vietnam.’ All studies for which there was access to the abstract and that had a principal hypothesis related to both diseases and/or any study of TB patients that reported individual data on DM, or *vice versa*, were included. Articles written in the English language were included and were grouped by country of origin. *A priori* topics of interest included regional DM/TB epidemiology, bi-directional screening and management strategies. Additionally, we selected large cohort TB-DM studies or reviews from other regions of the world to give an overall insight into the global TB-DM epidemiological situation. We also searched the available literature for the specific characteristics of DM in Asian populations and their potential role in increased susceptibility to M. tuberculosis infection.

## Results and Discussion

### Global TB-DM burden

In the last decade, a rapidly growing epidemic of DM in low- and middle-income countries and a slower than expected decline in global TB incidence rates have raised serious concerns [11]. In the Middle East, for example, the TB prevalence rate in 2010 was as low as 6.2 per 100,000 people in the United Arab Emirates, 71 per 100,000 people in Yemen, 51 per 100,000 people in Kuwait, and 23 per 100,000 people in Saudi Arabia and Iran [12]. However, with growing DM prevalence in these countries and a great number of expatriate workers coming from countries with a high prevalence of TB, TB-DM comorbidity has become a serious issue. In 2015, a systematic review identified 59 studies on DM and TB from 10 Middle East countries [13]. The prevalence of TB-DM comorbidity was very variable among studies (from 4.2% in Iran to 60% in Yemen). In Africa, DM prevalence among TB patients also varies greatly between studies (3.35–16.4%) [14–17]. HIV co-infection, age (older than 45), overweight, and being male were identified as predictive factors for TB-DM association [16,17]. There is also a high prevalence of TB in children and adolescents with DM1 in South Africa [18]. The studies that have evaluated the prevalence of TB-DM comorbidity in Hispanic populations all came to the same general conclusion: in this population, people affected by DM have 3 times the chance of developing TB compared to the non-diabetic population [6,19,20].

Even in developed countries, where TB is often largely controlled, a TB-DM association has been postulated, although the results also vary to a great degree. A cohort of 5,849 TB patients from Barcelona recruited between 2000 and 2013 reported that DM prevalence ranged from 4% to 7.2% each year [21]. In 2006, Jick et al. conducted a case-control study in the United Kingdom finding that the adjusted odd ratio (OR) of TB was 3.8 for patients with history of DM (*p* < 0.05), and confirming that DM is an independent risk factor for TB [22]. However, a case-control study from Denmark, as well as various studies from Australia and the US, found only modestly increased TB risk estimates associated with DM [23–25]. The prevalence of DM among TB patients in Japan is higher than in other developed countries (13.1%), probably due to the fact that Japan has a significantly larger elderly population, compared to that in the UK or the US (23% compared to 16% and 12%, respectively) [26].

### Quantitative estimate of DM cases among active TB patients in developing Asian countries

In a global estimate, it was found that out of all TB cases, 15% could be attributable to DM, with 40% of those cases coming from India and China [5].

Our search identified 33 studies on DM-TB epidemiology, representing 11 separate countries. A total of 27 studies addressed DM quantitative estimates among TB patients, whereas 6 studies addressed TB quantitative estimates in diabetic individuals. India contributed the most studies [8]. No published studies were found from Afghanistan, Laos, Cambodia, and Singapore.

Out of 27 studies that addressed DM prevalence in TB patients, 13 were cross-sectional, 10 were prospective cohort studies comparing DM/TB to non-DM/TB patients, 2 studies had case-control design, and the remainder were retrospective studies evaluating the demographic and clinical characteristics of TB-infected patients.

India leads the region with regard to DM prevalence among TB patients (in one study, as many as 54.1% of all TB patients were reported to have DM). DM prevalence among TB patients from studies included in this review varies from 6.3% to 54.1% (Table 1).

**Table 1.** Diabetes mellitus and tuberculosis epidemiology and country-specific citations

Country	Estimated TB prevalence as the rate per 100,000 population (range) [27]	Estimated DM prevalence as a percentage of the total population aged 20–79 years [28]	Percentage of DM prevalence among TB patients from studies included in this review
Bangladesh	404 (211–659)	8.3	37 (29)
China	89 (78–102)	9.8	5.05 (30), 6.3 (31), 12.4 (32), 16.2 (33), 19.9 (34), 27.9 (35)
India	195 (131–271)	9.3	14.7 (20), 14 (36), 25 (37), 25.3 (38), 29 (39), 33 (40), 35.5 (41), 54.1 (42)
Indonesia	647 (513–797)	6.5	13.2 (43), 14.8 (44)
Democratic People’s Republic of Korea	552 (150–1210)	4.4	20 (45)
Malaysia	135 (63–232)	17.9	17.7 (46), 28.5 (47), 30 (48)
Nepal	215 (102–369)	3.7	9.1 (49)
Pakistan	341 (285–402)	8.1	25.9 (50)
Sri Lanka	99 (51–164)	8.0	9 (51)
Thailand	236 (161–326)	7.1	16.3 (52), 23 (53)
Vietnam	198 (83–362)	6.0	8.8 (54)

### Quantitative estimate of active TB cases among DM patients in developing Asian countries

The prevalence of TB is 4 times greater in the DM population than in the general population [14,35,55]. Our study identified 6 studies that addressed quantitative estimates of active TB in diabetic individuals – 2 from China and 1 from each of the following countries: Bangladesh, Korea, Nepal, and Pakistan.

A prospective study conducted in Dhaka, Bangladesh in 2011 included 17,344 DM patients screened for pulmonary TB and found that the incidence of confirmed TB cases among DM patients was double the one observed in the general population (213.33/100,000) [56]. In China, TB case notification rates in screened DM patients were several times higher than those of the general population (774/100,000) [57]. In another prospective study conducted in China, the rate of TB diagnosis was almost 3 times higher than that found in the general population in Yunnan province [58].

A large prospective study carried out in Korea in a cohort of 331,601 DM patients showed TB case notification rates of 180 per 100,000 during a 3-year follow-up [59], whereas a small cross-sectional study conducted in Nepal showed that 8 out of 100 DM patients were positive for pulmonary TB [60]. A retrospective study conducted in Pakistan found that the prevalence of TB in diabetic patients was 11.9%, 10 times higher than in non-diabetic patients (1.7%, *p* < 0.05) [61].

## Bi-directional screening in Asian countries with high TB-DM burden

Since India and China represent countries with some of the highest burdens of DM-TB in the world, the International Union Against Tuberculosis and Lung Disease (the Union) launched a project aiming to investigate the links between DM and TB through the implementation of bi-directional screening in selected health facilities in India and China. National stakeholder meetings between the Union, the World Health Organization (WHO), the World Diabetes Foundation (WDF), and national DM and TB authorities were held in China and India and standardized procedures of screening DM patients for TB and *vice versa*, a monitoring tool, and a quarterly system of reporting were developed. Forty health care providers from China and India were trained in bi-directional screening of DM and TB, data analysis, and writing scientific papers on DM and TB. In China, the implementation of the bi-directional screening programme started in September 2011 [32,57].

Our search identified 4 studies that specifically addressed bi-directional screening in patients with TB-DM comorbidity (Table 2).

**Table 2.** Bi-directional screening in high TB-DM burden Asian countries

Country	TB prevalence among DM patients from studies included in this review as the rate per 100,000 population	Percentage of DM prevalence among TB patients from studies included in this review
China Li et al. [32,57]	300–800	12.4
India India Diabetes Mellitus-Tuberculosis Study Group [62] Prakash et al. [63]	642–956 413	13 6.2

The Union is currently carrying out several projects similar to the one completed in India and China, aiming to develop primary care settings guidelines for screening of DM in TB patients and *vice versa*, and to train a great number of health care professionals for systemic screening. Such projects are ongoing in Pakistan and Indonesia.

Although it is obvious that bi-directional screening is highly needed in countries with TB-DM comorbidity, there are still many strategic issues to be resolved. One of the most important issues in developing countries is the cost-effectiveness of the screening. India and China are countries with a very high TB prevalence, but a large proportion of TB cases discovered in the studies in India and China were already diagnosed and on treatment prior to screening [64]. Thus, the cost-effectiveness of this approach has to be cautiously assessed. Furthermore, it is unclear whether screening should be directed at all patients or targeted at those with high-risk characteristics [64]. Although there is a large amount of data on the risk factors associated with TB-DM comorbidity, they are very ambiguous and further research is needed to identify populations at risk.

Another problem is related to the technologies for diagnosing TB and DM in routine settings. Fasting blood glucose testing was proved to have low sensitivity, whereas oral glucose tolerance test performed better as a screening tool when combined with HbAc1 [42]. TB diagnostic approaches that rely on sputum smear examination and chest radiography have low sensitivity as well [65]. Further work is needed to determine whether new TB and DM screening technologies, such as rapid nucleic acid amplification technology for TB diagnosis and autofluorescence-based readers or sudomotor function devices for DM screening, are feasible, more sensitive, and cost-effective.

The best time to screen TB patients for DM has not been defined either. Although screening at the time of registration would be easiest, stress-induced hyperglycaemia may lead to false-positive diagnoses. On the other hand, if the screening is performed at the end of the initial or continuation phase, the possibility of early DM intervention or TB treatment improvement will be lost [64].

Another issue in bi-directional screening is a lack of non-communicable disease programme or systematic, decentralized DM programme in these countries. Hence, it is very difficult to diagnose DM systematically, track what happens to the patient post-DM diagnosis, or implement TB screening among diabetic patients [64]. Given the escalating proportions of TB-DM comorbidity in Asian countries, more evidence is urgently needed to answer important questions about bi-directional screening in different settings.

## Sensitivity of Asian people with DM to TB

Although changes have been found in both innate and adaptive immune responses of people with DM, it is not clear why they are at increased risk of TB. The mechanisms underlying this susceptibility to TB are still in need of detailed investigation. In Asian people, DM has certain characteristics that may make this population more prone to TB in the presence of DM. These characteristics are probably a result of external (e.g. poor glycaemic control) and internal factors (e.g. specific mechanisms of insulin resistance, genetic susceptibility to DM).

### Poor glycaemic control

Achieving optimum glycaemic control is hard in Asian countries due to scarcity of adequate health care facilities, low educational status, and economic disparities. A 5-year survey, documenting changes in diabetes practice in developing regions that included 11,799 patients out of which 5,888 were Asian, showed that only 20–30% of patients were at the HbA1c < 7% goal [66]. The Diabcare-Asia project, a cross-sectional survey of 24,317 diabetic patients from Bangladesh, China, India, Indonesia, Malaysia, Philippines, Singapore, South Korea, Sri Lanka, Taiwan, Thailand, and Vietnam, found that 55% of patients had values of HbA1c exceeding 8% [67].

Poor glycaemic control in Asian populations represents a potentially important risk factor for TB. Restrepo and colleagues conducted one of the first studies which revealed that persistent hyperglycaemia could play a key role in altering the immune responses to M. tuberculosis in diabetic patients [68]. The study showed that poor DM control (as indicated by HbA1c level) was associated with differences in the innate and cellular cytokine responses to stimulation with purified protein derivative from M. tuberculosis, thus facilitating progression to active TB [68]. Another recent study, that recruited 4,690 elderly diabetic patients in Hong Kong, showed that the patients with greater HbA1c value (>7%) had a hazard risk of active TB that was 3 times increased compared with those who had HbA1c < 7% (HR 3.11; 95% CI 1.63–5.92, *p* < 0.01) [69]. A cohort study with 123,546 individuals performed in Taiwan found that, during a median follow-up period of 4.6 years, diabetic patients with poor glycaemic control had a significantly higher hazard risk of TB (adjusted HR 2.21; 95% CI 1.63–2.99, *p* < 0.01) compared to those without DM [70]. The evidence of poor glycaemic control in Asian patients, along with the fact that poor glycaemic control represents an important risk factor for TB, call for further therapeutic actions in order to decrease TB-DM prevalence in developing Asian countries.

### Insulin resistance

A very high prevalence of insulin resistance in Asian populations may contribute to susceptibility of this population to TB. It is known that TB affects the production of insulin and that it is detrimental to insulin sensitivity [71]. Although several studies have shown the role of insulin in cellular metabolism and phagocytosis of M. tuberculosis, little is known about insulin resistance as a potential risk factor for active TB [72,73]. Chao et al. investigated the immunological mechanisms underlying the susceptibility of diabetic patients to TB [74]. They measured the level of resistin, a protein produced by immune cells in humans that causes insulin resistance and inhibits reactive oxygen species (ROS) production in leukocytes, and found that serum resistin levels were significantly higher in patient groups with severe TB and DM when compared with mild TB cases and healthy controls. They postulated that the elevation of serum resistin suppresses the Mycobacterium-induced immune response, leading to ineffective control of bacterial growth [74]. In the article by Mao et al., the authors hypothesize that functional changes in macrophages seen in the state of insulin resistance may increase the risk for active TB. They based their hypothesis on data that suggested insulin signalling may play a role in regulating the carbohydrate metabolism, redox activity, and phagocytic capacity in macrophages, that act as the first line of defence against M. tuberculosis invasion [75].

Insulin resistance is reported even in children and adolescents of Asian Indian origin [76]. Normally, insulin resistance is associated with visceral and subcutaneous fat content. However, characteristics of insulin resistance such as hyperinsulinaemia and hypertriglyceridaemia are seen even in non-obese Asian populations, especially in Asian Indian groups [76]. In the study of Chandalia et al., young men of south Asian origin who did not have increased intraperitoneal fat mass still had insulin resistance, unlike their white counterparts [77]. These findings suggest that the mechanisms underlying insulin resistance in these populations differ from those seen in white populations. More studies are needed to discover those mechanisms and reveal the role of insulin resistance in pathogenesis of TB.

### Genetic make-up

It is striking that even when they are not in their home countries, people of Asian or Indian/subcontinental origin show a higher prevalence of DM in the presence of TB than the rest of the population. A study examining 4 ethnic groups in England – white, black, ‘Asian’ (people from the subcontinent), and other (which included Chinese) – found 3,461 new cases of pulmonary TB during 2005. Of those, 384 (11.1%) were attributable to DM. The group most affected from both diseases was that of patients of subcontinental and Asian origin (55%). Black and white populations were affected in almost equal proportions (22% and 23%). The authors concluded that about one-third of Asians with newly diagnosed TB will have DM [78]. Similarly, Suwanpimolkul et al. gathered information from a TB clinic in San Francisco from 2005 to 2012 [79]. They recruited 791 patients with TB, of which 29.2% were born in the US, 26.7% in Asia, 11% in Mexico, and the remaining 33.1% were from other countries. Calculated prevalence of DM among patients with TB was 15.9%. Of these, 26.7% were patients of Asian origin (China and Philippines), accounting for the highest percentage of the non-Americans affected by both diseases [79]. These data suggest that there is something in the genetic make-up, or in early childhood, which makes people from these regions particularly sensitive to TB-DM comorbidity.

Although the possible genetic basis of high TB-DM susceptibility in Asians remains unclear, suggested genetic causes of predisposition to insulin resistance and DM in Asians may partially explain a higher prevalence of TB-DM in this group. Radha et al. investigated the prevalence of the peroxisome proliferator-activated receptor (PPAR)-gamma Pro12Ala polymorphism (known to have a protective role against diabetes) in Caucasians, in a migrant population of South Asians, and in a homogeneous population of South Asians residing in India [80]. In all 3 populations, the authors observed a similar prevalence of the 12Ala allele. However, while the frequency of this allele was significantly lower in the diabetic Caucasians when compared with the non-diabetic Caucasians, South Asian diabetic and non-diabetic subjects had virtually the same prevalence of 12Ala allele [80]. This finding suggests that PPAR-gamma Pro12Ala polymorphism is protective against DM in Caucasians but not in South Asians. Chang et al. identified a novel risk-conferring genetic variant of the transcription factor 7-like 2 (TCF7L2) for DM2 in a Chinese population, different from the variants observed in populations of European ancestry [81]. Further studies are needed to discover whether these genetic polymorphisms, specific to Asian populations, are responsible for higher rates of DM and insulin resistance and consequently for higher susceptibility to TB in this patient group.

### Age

A high prevalence of TB-DM comorbidity in Asian countries, especially in China and India, may be a result of the rapidly increasing prevalence of young-onset DM in these countries. In southern India, from 2000 to 2006, the prevalence of DM in people younger than 44 years increased by 10.7% [82]. Data from China show an 88% increase in the prevalence of maturity-onset DM in the 35–44 years age group from 1994 to 2000, probably due to the rapid transition in dietary habits, reduced physical activity, longer working hours, and decreasing sleep hours [82]. Although the data is very ambiguous, it has been shown that the relation between DM and TB is more prominent in younger people. A meta-analysis of 13 observational studies of association between DM and active TB identified 2 studies that presented age-stratified RRs (Risk ratios). They demonstrated stronger associations of DM and TB under the age of 40 and declining RR with increasing age in age groups over 40 years (trend pKim = 0.014, pPonce-de-Leon = 0.184) [83]. However, since many other studies found that TB-DM comorbidity was significantly more common in patients over 40 years [31,35,36], these results and the potential relation of young-onset DM and TB should be taken with caution.

## Treatment challenges and current TB-DM comorbidity care guidelines in Asian countries with high TB-DM burden

In general, the clinical course of a patient with both TB and DM tends to be more severe due to factors like immunosuppression, different pharmacokinetics of anti-TB and DM drugs, and comorbidities that affect the clinical course, such as hypertension and obesity [71,84,85]. Treatment failure occurs in 4.8% of TB-DM patients compared with a failure rate of 1.5% in non-diabetic TB patients [86]. Furthermore, the TB relapse rate (20%) and multidrug-resistant TB frequency (17.7%) are statistically significantly higher in DM patients compared with non-DM (5.3% and 8.4%, respectively). Although the implementation of the Directly Observed Treatment Short course (DOTs) strategy has been widely accepted in developing Asian countries, there are still many uncertainties related to the optimum treatment strategies in patients with both TB and DM.

Some of the most important problems are the length of anti-TB treatment, drug–drug interactions (e.g. rifampicin and oral sulphonylurea derivatives), drug–disease interactions (e.g. peripheral neuropathy induced by both isoniazid and DM), adherence to medication being compromised by high pill counts or adverse drug effects, and the lack of DM clinics designed to prevent easy M. tuberculosis transmission [87]. In addition to these issues, Asian countries face insufficient supplies of second-line anti-TB drugs and shortages of trained personnel [88].

Currently, there is insufficient evidence to support changing the recommended standard TB treatment regimens or making specific recommendations for clinical case management of TB in people with DM. It is common clinical practice in some settings to extend the duration of TB treatment in these patients. However, there are very few published trials on the effectiveness of extending the duration of treatment. In Asian populations, only 1 study specifically addressed the question of optimal duration of anti-TB treatment in patients with DM. The study of Wang et al. classified 12,688 Taiwanese TB patients with DM in 6-month and 9-month anti-TB treatment groups [89]. They concluded that extending the anti-TB treatment by 3 months may decrease the recurrence rate when the DOTs strategy is not applied. In a retrospective population-based study that included 201 newly diagnosed pulmonary TB patients in Shanghai, Wu et al. found that pulmonary TB patients with DM more frequently had an extended anti-TB treatment duration than those without DM, but they could not confirm that the similarity of treatment outcomes was a result of the extended treatment duration [34].

A multi-centre cohort study conducted in China in 2015 compared treatment success rates between pulmonary TB patients with DM who received standardized anti-TB treatment and a group of pulmonary TB patients with DM who received an individualized, drug susceptibility testing (DST)-based treatment [90]. The authors reported higher treatment success rates in the individualized treatment group than in the conventionally treated group (83.3% vs. 60.0% respectively, *p* = 0.045).

According to the WHO recommendations (2011), the same TB treatment regimen should be prescribed to people with DM as for people without it, whereas the management of DM in TB patients should be provided in line with special recommendations for people with worsened glucose control as an effect of an ongoing infection [91]. China’s National TB programme guidelines recommend that all patients with TB should receive the standardized short-term regime for TB treatment, except those with MDR-TB [92]. However, there is insufficient information about how TB-DM patients would respond to this short treatment regime, as good or poor control of DM in patients may lead to different treatment outcomes [93,94]. Technical and operational guidelines issued by the Central Tuberculosis Division of the Indian government recommend the standardized short-term regime for TB treatment for all patients, except for those with MDR-TB and central nervous system, skeletal, or disseminated drug-sensitive TB. This document includes specific guidelines for special patient groups such as pregnant patients, those with seizures, psychosis, or renal or hepatic impairment, but not for patients with DM [95]. A comprehensive guideline for the management of TB-DM comorbidity developed by the WHO and Cambodia Diabetes Association (CDA) also applies WHO recommendations on TB management, advising the use of insulin, metformin, sulphonylurea derivatives, and thiazolidinediones in patients with dual disease [96].

There are not yet randomized trials that evaluate an adequate treatment for TB-DM, neither there is sufficient evidence on drug–drug and drug–disease interactions in the TB-DM patient group. A systematic review aiming to determine the effect of insulin, alone or in combination with oral hypoglycaemic agents, and the effect of poor glycaemic control on unsuccessful TB treatment outcome(s) is currently ongoing [97].

Optimal TB-DM management and integration of services could lead to the earlier start of treatment for DM and improved health outcomes for those with dual disease. More studies that address this topic are urgently needed and are especially important for developing Asian countries.

## Conclusions

The studies summarized from East, South, and South-East Asia demonstrated very inconsistent co-prevalence rates, with a prevalence of DM among TB patients ranging from approximately 5% to more than 50%. The data on the prevalence of TB among patients with DM are limited, but available evidence from China, Bangladesh, Korea, Nepal, and Pakistan shows a TB prevalence rate 1.8–9.5 times higher than in the general population. High prevalence rates of TB in Asian patients with DM are at least partially a consequence of poor glycaemic control in Asian patients and their high insulin resistance rates.

The majority of studies were retrospective analyses without a specific design to compare DM/TB and non-DM/TB patients, and while certain countries had numerous studies (China, India) there were 4 countries for which studies of DM/TB were not found (Afghanistan, Laos, Cambodia, and Singapore).

Developing Asian countries face many strategic issues, like insufficient supplies of second-line anti-TB drugs, shortages of trained personnel, and lack of DM facilities designed to prevent transmission of Mycobacteria. Additionally, we found a significant knowledge gap related to optimum TB-DM treatment strategies, and drug–disease and drug–drug interactions in this area. Our search identified only 1 study specifically designed to compare the extended anti-TB regime in diabetic patients and only 1 guideline developed specifically for TB-DM care.

Although we identified several studies that specifically addressed the implementation of bi-directional screening, they were limited to 2 countries only (India and China) and there were no studies that examined the best method or could comment on the diagnostic yield.

There exists considerable potential for meaningful study of DM/TB in the region. Prospective studies specifically designed to address differences between DM and non-DM TB patients, interpretation of DM/TB treatment outcomes, drug–drug and drug–disease interactions, and the optimal TB and DM diagnostic strategies are urgently needed. Decentralized DM care facilities would provide a systematic DM diagnosis and may prevent easy M. tuberculosis transmission. Defining the population at risk followed by bi-directional screening implementation in the whole region should be the ultimate goal of the health authorities.

## Paper context

Asia faces an epidemic of TB-DM comorbidity, but its prevalence in the South, East, and South-East regions remains under-examined. Along with bi-directional screening and optimal treatment issues, we detected very inconsistent co-prevalence rates, and an important knowledge gap related to screening and TB-DM management. Prospective studies specifically designed to address differences between DM and non-DM TB patients, interpretation of DM/TB treatment outcomes, best screening methods and strategy, together with systematic DM programme implementation are urgently needed.

## References

[CIT0001] WHO (2016). Global report on diabetes 2016.

[CIT0002] Lu B, Zhang S, Wen J (2010). The New Unified International Diabetes Federation/American Heart Association/National Heart, Lung, and Blood Institute Metabolic Syndrome definition: does it correlate better with C-reactive protein in Chinese patients diagnosed with type 2 diabetes?. J Int Med Res.

[CIT0003] WHO (2015). Global tuberculosis report 2015.

[CIT0004] WHO (2015). Implementing the end TB strategy: the essentials.

[CIT0005] Lönnroth K, Roglic G, Harries AD. (2014). Improving tuberculosis prevention and care through addressing the global diabetes epidemic: from evidence to policy and practice. Lancet Diabetes Endocrinol.

[CIT0006] Ruslami R, Aarnoutse RE, Alisjahbana B (2010). Implications of the global increase of diabetes for tuberculosis control and patient care. Trop Med Int Health.

[CIT0007] Harries AD, Lin Y, Satyanarayana S (2011). The looming epidemic of diabetes-associated tuberculosis: learning lessons from HIV-associated tuberculosis. Int J Tuberc Lung Dis.

[CIT0008] WDF (2009). Screening for diabetes in TB patients WDF08-380.

[CIT0009] Muruganathan A, Viswanathan V, Wishwanathan M (2016). The double burden of tuberculosis and diabetes in India. Diabetology. Complications of diabetes.

[CIT0010] WDF (2011). Improving diabetes screening among tuberculosis patients WDF12-621.

[CIT0011] Harries AD, Satyanarayana S, Kumar AM (2013). Epidemiology and interaction of diabetes mellitus and tuberculosis and challenges for care: a review. Public Health Action.

[CIT0012] WHO (2011). Global health statistics 2011.

[CIT0013] Alkabab YM, Al-Abdely HM, Heysell SK (2015). Diabetes-related tuberculosis in the Middle East: an urgent need for regional research. Int J Infect Dis.

[CIT0014] Workneh MH, Bjune GA, Yimer SA (2016). Prevalence and associated factors of diabetes mellitus among tuberculosis patients in South-Eastern Amhara Region, Ethiopia: a cross sectional study. PLoS One.

[CIT0015] Balde NM, Camara A, Camara LM (2006). Associated tuberculosis and diabetes in Conakry, Guinea: prevalence and clinical characteristics. Int J Tuberc Lung Dis.

[CIT0016] Faurholt-Jepsen D, Range N, PrayGod G (2012). The role of anthropometric and other predictors for diabetes among urban Tanzanians with tuberculosis. Int J Tuberc Lung Dis.

[CIT0017] Kibirige D, Ssekitoleko R, Mutebi E (2013). Overt diabetes mellitus among newly diagnosed Ugandan tuberculosis patients: a cross sectional study. BMC Infect Dis.

[CIT0018] Webb EA, Hesseling AC, Schaaf HS (2009). High prevalence of Mycobacterium tuberculosis infection and disease in children and adolescents with type 1 diabetes mellitus. Int J Tuberc Lung Dis.

[CIT0019] Pablos-Méndez A, Blustein J, Knirsch CA (1997). The role of diabetes mellitus in the higher prevalence of tuberculosis among Hispanics. Am J Public Health.

[CIT0020] Stevenson CR, Forouhi NG, Roglic G (2007). Diabetes and tuberculosis: the impact of the diabetes epidemic on tuberculosis incidence. BMC Public Health.

[CIT0021] Moreno-Martínez A, Casals M, Orcau À (2015). Factors associated with diabetes mellitus among adults with tuberculosis in a large European city, 2000–2013. Int J Tuberc Lung Dis.

[CIT0022] Jick SS, Lieberman ES, Rahman MU (2006). Glucocorticoid use, other associated factors, and the risk of tuberculosis. Arthritis Rheum.

[CIT0023] Leegaard A, Riis A, Kornum J (2011). Diabetes, glycemic control, and risk of tuberculosis: a population-based case-control study. Diabetes Care.

[CIT0024] Marks G, Dobler C, Flack J (2009). Estimation of the risk of tuberculosis among people with diabetes. Eur Respir J.

[CIT0025] Marks SM (2011). Diabetes and tuberculosis, US National Health Interview Survey, 2000–2005. Int J Tuberc Lung Dis.

[CIT0026] Uchimura K, Ngamvithayapong-Yanai J, Kawatsu L (2014). Characteristics and treatment outcomes of tuberculosis cases by risk groups, Japan, 2007–2010. West Pac Surveill Response J.

[CIT0027] WHO (2016). Tuberculosis country profiles.

[CIT0028] WHO (2016). Diabetes country profiles.

[CIT0029] Tofaz T (2016). A survey on the prevalence of diabetes in tuberculosis patients in NIDCH hospital, Dhaka.

[CIT0030] Xiao X, Zhou C, Jiang W (2015). Demographic distribution of diabetes–tuberculosis comorbidity in Eastern rural China. Int J Mycobacteriol.

[CIT0031] Wang Q, Ma A, Han X (2013). Prevalence of type 2 diabetes among newly detected pulmonary tuberculosis patients in China: a community based cohort study. PLoS One.

[CIT0032] Li L, Lin Y, Mi F (2012). Screening of patients with tuberculosis for diabetes mellitus in China. Trop Med Int Health.

[CIT0033] Hongguang C, Min L, Shiwen J (2015). Impact of diabetes on clinical presentation and treatment outcome of pulmonary tuberculosis in Beijing. Epidemiol Infect.

[CIT0034] Wu Z, Guo J, Huang Y (2016). Diabetes mellitus in patients with pulmonary tuberculosis in an aging population in Shanghai, China: prevalence, clinical characteristics and outcomes. J Diabetes Complications.

[CIT0035] Ko P-Y, Lin S-D, Tu S-T (2016). High diabetes mellitus prevalence with increasing trend among newly-diagnosed tuberculosis patients in an Asian population: a nationwide population-based study. Prim Care Diabetes.

[CIT0036] Khanna A, Lohya S, Sharath BN (2013). Characteristics and treatment response in patients with tuberculosis and diabetes mellitus in New Delhi, India. Public Health Action.

[CIT0037] Jagran Pehel (2015). http://www.jagranpehel.com/ContentPages/News/NewsDetails.aspx?newsId=198.

[CIT0038] Viswanathan V, Kumpatla S, Aravindalochanan V (2012). Prevalence of diabetes and pre-diabetes and associated risk factors among tuberculosis patients in India. PLoS One.

[CIT0039] Raghuraman S, Vasudevan KP, Govindarajan S (2014). Prevalence of diabetes mellitus among tuberculosis patients in urban Puducherry. N Am J Med Sci.

[CIT0040] Nair S, Kumari AK, Subramonianpillai J (2013). High prevalence of undiagnosed diabetes among tuberculosis patients in peripheral health facilities in Kerala. Public Health Action.

[CIT0041] Jali MV, Mahishale VK, Hiremath MB (2013). Diabetes mellitus and smoking among tuberculosis patients in a tertiary care centre in Karnataka, India. Public Health Action.

[CIT0042] Kornfeld H, West K, Kane K (2016). High prevalence and heterogeneity of diabetes in patients with TB in South India: a report from the Effects of Diabetes on Tuberculosis Severity (EDOTS) study. Chest.

[CIT0043] Alisjahbana B, van Crevel R, Sahiratmadja E (2006). Diabetes mellitus is strongly associated with tuberculosis in Indonesia. Int J Tuberc Lung Dis.

[CIT0044] Alisjahbana B, Sahiratmadja E, Nelwan EJ (2007). The effect of type 2 diabetes mellitus on the presentation and treatment response of pulmonary tuberculosis. Clin Infect Dis.

[CIT0045] Oh KH, Kim HJ, Kim MH (2016). Non-communicable diseases and risk of tuberculosis in Korea. Int J Tuberc Lung Dis.

[CIT0046] Ismail Y (2004). Pulmonary tuberculosis – a review of clinical features and diagnosis in 232 cases. Med J Malaysia.

[CIT0047] Elamin E, Muttalif A, Mohamed Ibrahim MI (2004). A survey on tuberculosis cases in Penang hospital: preliminary findings. Mal J Pharmaceutical Sci.

[CIT0048] Syed Suleiman SA, Ishaq Aweis D, Mohamed A (2012). Role of diabetes in the prognosis and therapeutic outcome of tuberculosis. Int J End.

[CIT0049] Thapa B, Paudel R, Thapa P (2015). Prevalence of diabetes among tuberculosis patients and associated risk factors in Kathmandu valley. Saarc J Tuber Lung Dis Hiv/Aids.

[CIT0050] Usmani RA, Nasir MI, Wazir S (2014). Diabetes mellitus among tuberculosis patients in a tertiary care hospital of Lahore. J Ayub Med Coll Abbottabad.

[CIT0051] Rajapakshe W, Isaakidis P, Sagili KD (2015). Screening patients with tuberculosis for diabetes mellitus in Ampara, Sri Lanka. Public Health Action.

[CIT0052] Duangrithi D, Thanachartwet V, Desakorn V (2013). Impact of diabetes mellitus on clinical parameters and treatment outcomes of newly diagnosed pulmonary tuberculosis patients in Thailand. Int J Clin Pract.

[CIT0053] Reechaipichitkul W, So-Ngern A, Chaimanee P (2014). Treatment outcomes of new and previously-treated smear positive pulmonary tuberculosis at Srinagarind Hospital, a tertiary care center in northeast Thailand. J Med Assoc Thai.

[CIT0054] Thanh NP, Khue PM, Sy DN (2015). Diabetes among new cases of pulmonary tuberculosis in Hanoï, Vietnam. Bull Soc Pathol Exot.

[CIT0055] Arnold M, Beran D, Haghparast-Bidgoli H (2016). Coping with the economic burden of diabetes, TB and co-prevalence: evidence from Bishkek, Kyrgyzstan. BMC Health Serv Res.

[CIT0056] Rahim Z, Momi MS, Saha SK (2012). Pulmonary tuberculosis in patients with diabetes mellitus in Bangladesh. Int J Tuberc Lung Dis.

[CIT0057] Lin Y, Li L, Mi F (2012). Screening patients with diabetes mellitus for tuberculosis in China. Trop Med Int Health.

[CIT0058] Lin Y, Innes A, Xu L (2015). Screening of patients with diabetes mellitus for tuberculosis in community health settings in China. Trop Med Int Health.

[CIT0059] Heo EY, Choi N-K, Yang BR (2015). Tuberculosis is frequently diagnosed within 12 months of diabetes mellitus. Int J Tuberc Lung Dis.

[CIT0060] Regmi HS, Gurung R, Sharma SK (2014). Pulmonary tuberculosis among diabetic patients in Dharan Municipality, Eastern Nepal. Int J Infect Dis.

[CIT0061] Jabbar A, Hussain SF, Khan AA (2014). Clinical characteristics of pulmonary tuberculosis in adult Pakistani patients with co-existing diabetes mellitus. La Revue de Santé de la Méditerranée Orientale.

[CIT0062] India Diabetes Mellitus-Tuberculosis Study Group (2013). Screening of patients with diabetes mellitus for tuberculosis in India. Trop Med Int Health.

[CIT0063] Prakash BC, Ravish KS, Prabhakar B (2013). Tuberculosis-diabetes mellitus bidirectional screening at a tertiary care centre, South India. Public Health Action.

[CIT0064] Harries AD, Kumar AM, Satyanarayana S (2015). Diabetes mellitus and tuberculosis: programmatic management issues. Int J Tuberc Lung Dis.

[CIT0065] Ryu YJ (2015). Diagnosis of pulmonary tuberculosis: recent advances and diagnostic algorithms. Tub Resp Dis.

[CIT0066] Ji L-N, Lu J-M, Guo X-H (2013). Glycemic control among patients in China with type 2 diabetes mellitus receiving oral drugs or injectables. BMC Public Health.

[CIT0067] Diabcare-Asia 1998 Study Group (2002). The status of diabetes control in Asia – a cross-sectional survey of 24 317 patients with diabetes mellitus in 1998. Diabet Med.

[CIT0068] Restrepo B, Fisher-Hoch S, Pino P (2008). Tuberculosis in poorly controlled type 2 diabetes: altered cytokine expression in peripheral white blood cells. Clin Infect Dis.

[CIT0069] Leung CC, Lam TH, Chan WM (2008). Diabetic control and risk of tuberculosis: a cohort study. Am J Epidemiol.

[CIT0070] Lee P-H, Fu H, Lai T-C (2016). Glycemic control and the risk of tuberculosis: a cohort study. Plos Med.

[CIT0071] Baghaei P, Marjani M, Javanmard P (2013). Diabetes mellitus and tuberculosis facts and controversies. J Diabetes Metab Disord.

[CIT0072] Yano H, Kinoshita M, Fujino K (2012). Insulin treatment directly restores neutrophil phagocytosis and bactericidal activity in diabetic mice and thereby improves surgical site Staphylococcus aureus infection. Infect Immun.

[CIT0073] Lima AO, Queiroz M, Bräscher HM (1979). Experientia.

[CIT0074] Chao W-C, Yen C-L, Wu Y-H (2015). Increased resistin may suppress reactive oxygen species production and inflammasome activation in type 2 diabetic patients with pulmonary tuberculosis infection. Microbes Infect.

[CIT0075] Mao F, Chen T, Zhao Y (2011). Insulin resistance: a potential marker and risk factor for active tuberculosis?. Med Hypotheses.

[CIT0076] Ramachandran A, Wan Ma RC, Snehalatha C (2010). Diabetes in Asia. Lancet.

[CIT0077] Chandalia M, Lin P, Seenivasan T (2007). Insulin resistance and body fat distribution in South Asian men compared to Caucasian men. PLoS One.

[CIT0078] Walker C, Unwin N (2010). Estimates of the impact of diabetes on the incidence of pulmonary tuberculosis in different ethnic groups in England. Thorax.

[CIT0079] Suwanpimolkul G, Grinsdale JA, Jarlsberg LG (2014). Association between diabetes mellitus and tuberculosis in United States-born and foreign-born populations in San Francisco. PLoS One.

[CIT0080] Radha V, Vimaleswaran KS, Babu HN (2006). Role of genetic polymorphism peroxisome proliferator-activated receptor-gamma2 Pro12Ala on ethnic susceptibility to diabetes in South-Asian and Caucasian subjects: evidence for heterogeneity. Diabetes Care.

[CIT0081] Chang Y-C, Chang T-J, Jiang Y-D (2007). Association study of the genetic polymorphisms of the transcription factor 7-like 2 (TCF7L2) gene and type 2 diabetes in the Chinese population. Diabetes.

[CIT0082] Ramachandran A, Snehalatha C, Shett A (2012). Trends in prevalence of diabetes in Asian countries. World J Diabetes.

[CIT0083] Jeon CY, Murray MB (2008). Diabetes mellitus increases the risk of active tuberculosis: a systematic review of 13 observational studies. Plos Med.

[CIT0084] Baker MA, Harries AD, Jeon CY (2011). The impact of diabetes on tuberculosis treatment outcomes: a systematic review. BMC Med.

[CIT0085] Gil-Santana L, Almeida-Junior JL, Oliveira CA (2016). Diabetes is associated with worse clinical presentation in tuberculosis patients from Brazil: a retrospective cohort study. PLoS One.

[CIT0086] Ponce-De-Leon A, Garcia-Garcia MML, Garcia-Sancho MC (2004). Tuberculosis and diabetes in southern Mexico. Diabetes Care.

[CIT0087] Sullivan T, Amor Y (2012). The co-management of tuberculosis and diabetes: challenges and opportunities in the developing world. Plos Med.

[CIT0088] Institute of Medicine (US) (2012). Facing the reality of drug-resistant tuberculosis in India: challenges and potential solutions: summary of a joint workshop by the institute of medicine, the Indian National Science Academy, and the Indian Council of Medical Research.

[CIT0089] Wang J-Y, Lee M-C, Shu C-C (2015). Optimal duration of anti-TB treatment in patients with diabetes: nine or six months?. Chest.

[CIT0090] Du J, Gao W, Ma Y (2015). Treatment effect analysis of the standard regimen and the optimized regimen for retreatment pulmonary tuberculosis complicated with diabetes. Zhonghua Jie He He Hu Xi Za Zhi.

[CIT0091] WHO (2010). Treatment of tuberculosis: guidelines-fourth edition.

[CIT0092] Chinese Center for Disease Control and Prevention (2009). Disease Control Bureau and Medical Administrative Department of Ministry of Health of the People’s Republic of China. National Tuberculosis Control Programme Guideline in China (2008).

[CIT0093] Mi F, Jiang G, Du J (2014). Is resistance to anti-tuberculosis drugs associated with type 2 diabetes mellitus? A register review in Beijing, China. Glob Health Action.

[CIT0094] Mi F, Tan S, Liang L (2013). Diabetes mellitus and tuberculosis: pattern of tuberculosis, two-month smear conversion and treatment outcomes in Guangzhou, China. Trop Med Int Health.

[CIT0095] Central TB division (2016). Ministry of Health & Family Welfare – Government of India. Technical and operational guidelines for TB control in India.

[CIT0096] World Health Organization and Cambodia Diabetes Association (2014). Draft guideline for management of diabetes mellitus-tuberculosis comorbidity. http://webcache.googleusercontent.com/search?q=cache:nuIRNFnlNM0J:worlddiabetesfoundation.org/sites/default/files/WDF14-852%2520Draft%2520Guidelines%2520TB-DM%252007-01-2015.docx+&cd=1&hl=en&ct=clnk&gl=rs.

[CIT0097] Shewade HD, Jeyashree K, Mahajan P (2016). Effect of insulin, with or without oral hypoglycemic agents, (compared to oral hypoglycemic agents only) and glycemic control, stringent or not stringent, (compared to no glycemic control) on unsuccessful TB treatment outcomes among patients with TB-diabetes: a systematic review. PROSPERO.

